# The Effect of Peritoneal Fluid from Patients with Endometriosis on Mitochondrial Function and Development of Early Mouse Embryos

**DOI:** 10.1371/journal.pone.0082334

**Published:** 2013-12-26

**Authors:** Jing Shu, Lili Xing, Guolian Ding, Qiong Luo, Xinmei Liu, Qingfeng Yan, Jianzhong Sheng, Hefeng Huang

**Affiliations:** 1 Department of Reproductive Endocrinology, Zhejiang Provincial People's Hospital, Hangzhou, PR China; 2 Women's Hospital, School of Medicine, Zhejiang University, Hangzhou, PR China; 3 Institute of Genetics, College of Life Science, Zhejiang University, Hangzhou, PR China; 4 School of Medicine, Zhejiang University, Hangzhou, PR China; Institute of Zoology, Chinese Academy of Sciences, China

## Abstract

**Background:**

Peritoneal fluid (PF) from patients with endometriosis can inhibit early embryo development via probable functional changes of embryo mitochondria in the early stage of embryo development. The purpose of this study was to determine the effect of PF from patients with endometriosis on mitochondrial function and development of early mouse embryos.

**Methodology/Principal Findings:**

PF was collected from patients with infertility and endometriosis, infertility due to tubal factors, and normal control subjects, and the level of NO was measured. Early murine embryos were then cultured with PF from normal control subjects, those with endometriosis, and with human tubal fluid (HTF), respectively. Cleavage and blastulation rates, mitochondrial DNA (mtDNA) copy numbers, adenosine triphosphate (ATP) level, and mitochondrial membrane potential (ΔΨm) of the different groups were compared. The NO level in the PF of patients with endometriosis was significantly greater than in those without endometriosis and control patients. The embryos cultures with PF from patients with endometriosis had a lower cleavage rate and blastulation rate, and higher ATP and ΔΨm level at the 2- and 4-cell stages. No significant difference was found in mtDNA copies among the 3 groups.

**Conclusions/Significance:**

PF from patients with endometriosis can inhibit early embryo development via probable functional changes of embryo mitochondria in the early stage of embryo development. Understanding the effects of PF on embryo development may assist in developing new methods of treatment for infertility.

## Introduction

Epidemiological studies have shown that endometriosis is strongly associated with infertility; however, the exact mechanism of endometriosis-induced infertility remains unclear [1]–[3]. While severe endometriosis complicated by pelvic anatomical structural changes can lead to impaired sperm and ovum transport, which may explain the concurrent infertility, mild endometriosis without pelvic anatomical abnormalities also often causes infertility [4], [5].

In patients with mild endometriosis there is an increase in the amount of pelvic fluid [6]. Pelvic peritoneal fluid (PF) is the complex of fallopian tubal fluid and peritoneal and ovarian secretions which contains a variety of cellular and non-cellular components. These fluids encircle the ovaries and can penetrate into the fallopian tube cavity, forming the microenvironment for sperm-egg fertilization and embryo development. Significant immune activity changes are present in the PF of patients with endometriosis [7], which is characterized by increases in a variety of cytokines, interleukins, and reactive oxygen species (ROS) [8], [9]. These changes result in a markedly deteriorated microenvironment for the early embryo. However, it is still inconclusive whether PF in patients with endometriosis really damages the quality of eggs and embryos [10]–[14].

Similar to ROS, reactive nitrogen species (RNS) are hazardous components that can cause mitochondrial damage. In patients with endometriosis, high levels of nitric oxide (NO) and nitric oxide synthase (NOS) can be detected in ectopic and eutopic endometrium [15]. NO can interact with compounds such as ROS producing free radicals and nitro compounds with high oxidation activity such as peroxynitrite anion (ONOO•) and peroxynitrite (HOONO). RNS can damage biological macromolecules, and high concentrations of NO can damage sperm, eggs, and embryos. While early studies indicated that NO content was not increased in the PF of patients with endometriosis [16], more recent studies have shown an increased concentration of NO in the PF of patients with endometriosis [17], [18]. Furthermore, a recent study by Polak et al. [19] showed that levels of the oxidative stress markers 8-hydroxy-2-deoxyguanosine (8-OHdG) and 8-isoprostane were increased in the PF of patients with endometriosis.

Mitochondria are important organelles that have functions including ATP synthesis, regulation of cellular calcium homeostasis and oxidative balance, and mediation of signal transduction. In addition, mitochondria have functions related to fertilization and embryo growth and differentiation [20]. Mitochondria are extremely sensitive to the environment, and they are affected by a variety of free radicals, ROS, and RNS. At the early stage of embryonic development, the zygotic genome is not activated and there is a lack of mitochondrial replication and renovation [21], thus, alterations in the embryonic microenvironment may cause serious consequences.

The purpose of this study was to examine the levels of NO in the PF of patients with endometriosis, and to determine the effects of PF from patients with endometriosis on mitochondria and the development of mouse embryos.

## Materials and Methods

This study consisted of two parts, a clinical study and an animal study. The clinical study was designed to measure the level of NO in the PF of patients with infertility due to endometriosis, tubal factor infertility, and subjects with normal fertility to determine the association of PF NO with endometriosis and infertility. The animal study was performed to compare the effects of PF from normal control subjects, those with endometriosis, human tubal fluid (HTF), and sodium nitroprusside (SNP), respectively, on early embryo development and mitochondrial development in a murine model.

### Clinical Study

#### Subjects and peritoneal fluid collection

This study was approved by the Ethics Committee of School of Medicine, Zhejiang University, Hangzhou, China, and all patients provided written informed consent. Patients seen for infertility at the Affiliated Obstetrics and Gynecology Hospital, Zhejiang University School of Medicine and Department of Obstetrics and Gynecology of the Affiliated Run Run Shaw Hospital were recruited.

Three groups of patients were included in the study: patients with primary infertility and endometriosis, patients with tubal infertility, and subjects with normal fertility (control group).

Inclusion criteria for patients with endometriosis were: 1) 25–35 years of age; 2) primary infertility; 3) normal ovulation with a 26–32 days menstrual cycle; 4) normal semen test of partner; 5) hysterosalpingography showed bilateral tubal patency; 6) no pregnancy after three artificial insemination attempts; 7) laparoscopy-confirmed diagnosis of pelvic endometriosis (stage I/II, according to the American Society for Reproductive Medicine rFAS staging criteria).

Inclusion criteria for patients with tubal infertility were: 1) 25–35 years of age; 2) normal ovulation with a 26–32 days menstrual cycle; 3) normal semen test of partner; 4) laparoscopy revealed chronic inflammation of the fallopian tubes.

Inclusion criteria for control patients were: 1) 25–35 years of age; 2) at least one naturally achieved pregnancy and delivery; 3) undergoing laparoscopy for mesosalpinx cysts or tubal ligation; 4) no pelvic adhesions and no endometriosis lesions at laparoscopy.

Exclusion criteria for all groups of patients were: 1) history of pelvic surgery; 2) had any surgery or received any hormones in the 3 months before the study; 3) any factors which can cause infertility other than tubal factors or endometriosis.

In all groups, laparoscopy was performed in the follicular phase of the menstrual cycle and fluid in the pouch of Douglas was aspirated before any other procedures. A portion of the peritoneal fluid (PF) sample was sent for the detection of NO, and the remainder was immediately centrifuged at 2000 rpm for 5 min at 4°C. The supernatant was collected and stored at −80°C. PF from patients with endometriosis was designated PF-E, from patients with tubal infertility PF-T, and from control patients PF-C.

#### Detection of PF NO

DAF-2 (4,5-diaminofluorescein) is a sensitive fluorescent indicator for the detection of NO. In the presence of oxygen, 4,5-diaminofluorescein (DAF-2) reacts with NO to yield a highly fluorescent reaction product, triazolofluorescein (DAF-2T), which is a sensitive fluorescent indicator for the detection of NO. The fluorescence is monitored using an Olympus inverted microscope with excitation and emission wavelengths of 488 and 515 nm, respectively. Briefly, a tube containing 100 µl of PF was examined and the background fluorescence (F0) was determined. Then, 5 µl of DAF-2 was added to the sample and the tube was incubated for 30 min. Fluorescence was then measured (F). The difference in values (F - F0) was defined as ΔF, and was an indicator of PF NO concentration.

### Animal Experiments

This study was approved by the Institutional Animal Care and Use Committee (IACUC) of School of Medicine, Zhejiang University, Hangzhou, China. Female (8–10 weeks of age; weight, 20–25 g) and male (12–13 weeks of age; weight, 30–35 g) Institute of Cancer Research (ICR) mice were used in the experiments. The mice were purchased from the Experimental Animal Center of Zhejiang University and raised using 14/10 light cycle (14 hours of light) at 24°C. The animals had free access to food and drinking water. Animals were treated according to standard guidelines for the use and care of laboratory animals. For all following experiments, minimum of 30 embryos per group were used in each experiment and the experiments were repeated at least five times. The method of sacrifice was done by intraperitoneal injection of overdose of 1% pentobarbital at 0.01 ml/g body weight of mice. After animals got profoundly anesthetized, cervical dislocation was applied to mice for euthanasia.

#### Effect NO on mouse embryo development and embryo mitochondria

Female mice received an intraperitoneal injection of 10 IU of pregnant mare serum gonadotropin (PMSG), followed by 10 IU of human chorionic gonadotropin (HCG) 48 hours later. After injection of HCG, 1 female mouse and 1 male mouse were housed together in 1 cage overnight. The mice were then sacrificed by cervical dislocation. The fallopian tubes were removed and placed in modified human tubal fluid (mHTF; Irvine Scientific, USA). The ampulla of the fallopian tube was pierced using the tip of the needle of a 1 ml sterile syringe to disperse the eggs and embryos, which were washed several times with mHTF and then observed under a light microscope.

The 2PN embryos were transferred and cultured in varying concentrations of SNP. SNP can decompose and release NO, thus it is used as a NO donor in experiments *in vitro*. The 2PN embryos were cultured in HTF (Irvine Scientific) containing 4 different concentrations of SNP: 0, 10^−8^, 10^−6^, and 10^−4^ M (n = 30 mouse embryos in each group). Embryonic development, cleavage rate, and blastocyst formation rate were determined.

As described above, 2PN embryos were collected and cultured in different media. The HTF-control group was cultured in HTF only. The PF non-endometriosis group (PF-control) was cultured in HTF containing 10% (volume ratio) of PF from five normal fertile women without endometriosis. The PF endometriosis group (PF-E) was cultured in HTF containing 10% (volume ratio) of PF from five infertile women with endometriosis. Embryonic development in each group, the cleavage rate, and blastocyst formation rate were observed. Two-cell and 4-cell embryos, morulas, and blastocysts were obtained for the detection of mitochondrial indicators.

#### ATP detection

In the presence of ATP, the fluorescence of luciferin as a result of the luciferase reaction is proportional to the concentration of ATP. ATP measurements were performed with the ATP Determination Kit (A22066, Molecular Probes (A22066); Life Technologies Corporation, Carlsbad, CA, USA). Changes of chemiluminescence were measured with a Thermo Scientific Luminoskan Ascent chemiluminescence plate reader (Thermo Fisher Scientific Inc., Waltham, Massachusetts, USA).

#### Embryo mitochondria DNA (mtDNA) copy number analysis by real time PCR

Each embryo used for mitochondrial DNA analysis was loaded individually and incubated in 20 µL of lysis buffer (50 mM Tris–HCl, pH 8.5; 0.1 mM ethylene diaminetetraacetic acid; 0.5% Tween-20; and 200 µg/mL of proteinase K) at 55°C for 20 min, followed by heat inactivation of proteinase K at 95°C for 10 min, then stored at −80°C before real time PCR.

Primers were designed according to the GenBank mtDNA standard sequence (GI: 33115104) for C57 mice by Shanghai Sangon Biological Engineering Technology Services Co. Forward primer: CGAAAGGACAAGAGAAATAGAG; reverse primer: GAACAAGGTTTTAAGTCTTACGCA. The mouse mtDNA 2567–2723 amplified fragment product was 157 bp.

The reaction system consisted of 5 µl SYBR, 0.2 µl forward primer (10 µM), 0.2 µl reverse primer (10 µM), 3 µl mtDNA template in DNA lysis buffer, and 1.6 µl distilled water.

Reaction conditions were: 94°C denaturation for 5 min; 94°C denaturation 30 s, 61°C annealing 30 s, 72°C extension 30 s, for a total of 40 cycles. The concentration of DNA was determined from the external standard calibration curve. The preparation of the external standard for mt DNA absolute quantification was as follows. PCR was performed with total DNA from 5 oocytes in a 25 µl mixture of 25 mM MgCl_2_ 3 µl, 10×PCR buffer 5 µl, 10 µM primer each 1 µl, 2.5 mM of each deoxynucleotide triphosphate (dNTP) 4 µl, and 5 U/µl Taq DNA polymerase 0.25 µl (TAKARA). The primers were the same as used for real-time PCR. The reaction conditions were: denaturation at 94°C for 5 min; 35 cycles at 94°C for 30 s, 50°C for 30 s, and 72°C for 30 s; and an extension step of 10 min at 72°C. The PCR product was purified, diluted in water, and quantified by a spectrophotometer (Eppendorf, Hamburg, Germany). The quality of the purified DNA was considered acceptable if the absorbance 260–280 nm ratio was between 1.8 and 2.0. It was assumed that 1 ng of 157 bp dsDNA product contained 5.83×10^9^ molecules. According to the OD260 measured value, an absolute copy number of purified mtDNA can be calculated. Serial dilutions were made to produce standards with a known number of templates.

#### Observation of mitochondrial membrane potential (ΔΨ_m_)

Embryos were placed in mHTF containing 10 ug/ml of the fluorescent dye JC-1 (Biovision, catalog number 1130-5). At a high ΔΨm state JC-1 polymerizes and fluoresces red, whereas at a low energy state the dye is a monomer and fluoresces green. After 15 minutes of culture the embryos were washed 3 times with m-HTF and immediately observed.

Observations of red and green fluorescence distribution and intensity were performed with a 2-photon confocal microscope (Olympus BX61W1-FV1000). The light excitation and emission wavelengths for red and green fluorescence were 559 nm and 572 nm, and 488 nm and 520 nm, respectively. Image J software was used to calculate the ratio of red to green fluorescence intensity (ΔΨm).

#### Electron microscopic observation of embryo mitochondria

A single embryo was placed in 2.5% glutaraldehyde, and fixed at 4°C for at least 2 hours. It was then washed twice with 0.1 M PBS, 10 minutes each time. Next, the embryo was fixed with 1% osmium tetroxide for 1 hour, and washed twice with 0.1 M PBS, 10 minutes each time. It was then stained with 2% uranyl acetate for 30 minutes, and dehydrated in gradient ethanol (50%, 70%, and 90%, 10 minutes each). Next, it was dehydrated by 100% ethanol for 15 minutes with oscillation. It was then dehydrated twice with 100% acetone, 15 minutes each time, and soaked in 1∶1 acetone/Epon812 for 1.5 hours. The egg was then soaked in pure embedding medium for 1.5 h, and embedded. Then it was placed in an aggregator (37°C, 24 h; 45°C, 24 h; 60°C, 48 h), followed by preparation of ultrathin sections (120 nm). The slices were stained with 4% uranyl acetate for 20 minutes and with lead citrate for 5 minutes. The egg was observed under a TECNAI -10 Philips electron microscope (80–100 KV) and photographed.

### Statistical analyses

Normally distributed continuous variables were compared by one-way analysis of variance (ANOVA). When a significant difference between groups was apparent, multiple comparisons of means were performed using the Least Significant Difference (LSD) method. Data were presented as means ± standard deviation (SD). Kruskal-Wallis test was applied and data presented as median and inter-quartile range if the data was not normally distributed. All statistical assessments were two-sided and evaluated at the 0.05 level of significant difference. Statistical analyses were performed using SPSS 15.0 statistics software (SPSS Inc., Chicago, IL).

## Results

### Clinical study

There were 115 patients with a laparoscopic diagnosis of pelvic endometriosis (PF-E group) and 120 patients with tubal infertility (PF-T group) included in the analysis. The PF-control group consisted of 30 patients with a normal reproductive history who underwent laparoscopic surgery due to a mesosalpinx cyst or for tubal ligation. There was no significant difference of age among the 3 groups (*P* = 0.814). In the PF-E, PF-control, and PF-T groups, the NO levels in PF were 19.0±5.7, 11.7±3.6, and 12.2±3.6, respectively, and the PF volumes were 17.9±5.7 mL, 7.9±3.7 mL, and 8.4±3.7 mL, respectively. The PF volume and NO level of the PF-E group were both significantly greater than those of the PF-control and PF-T groups (all, *P*<0.001).

### Animal studies

#### Effects of SNP and PF on embryo development

**Figure 1:**
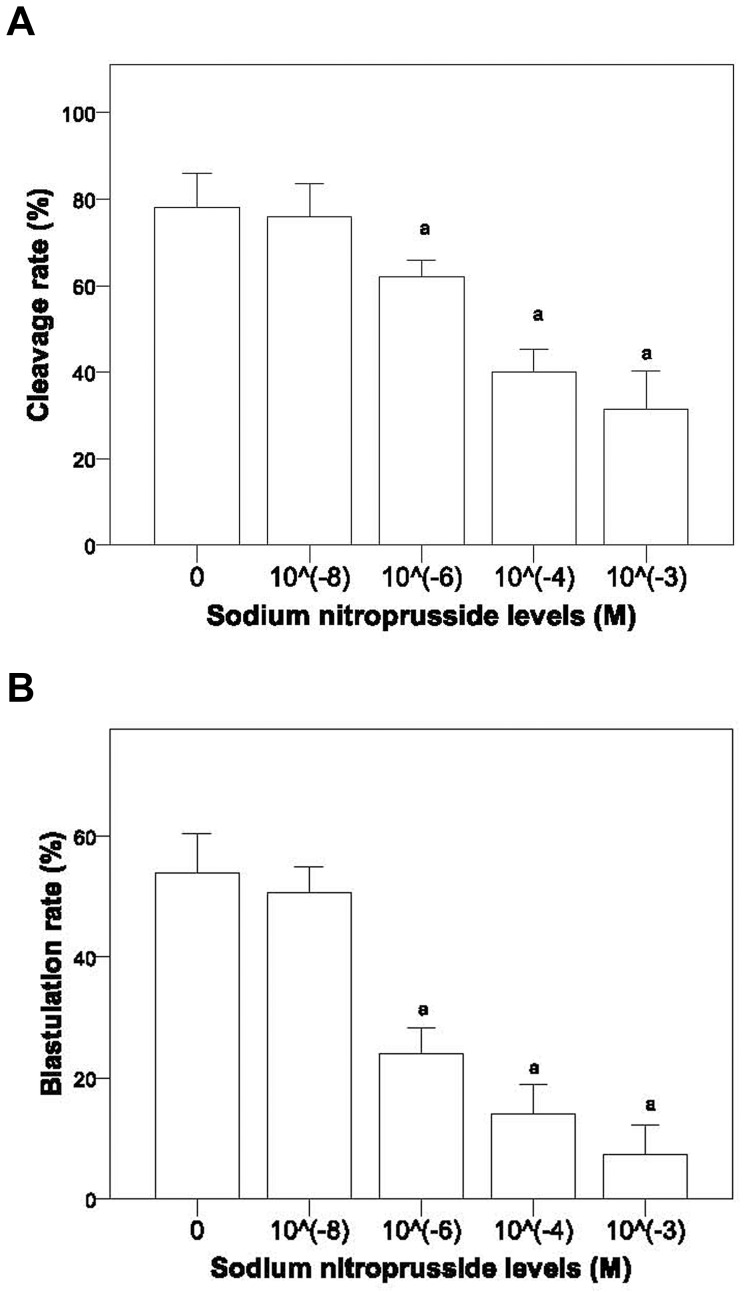
Cleavage (A) and blastulation (B) rates at different concentrations of sodium nitroprusside. ^a^ Indicates a significant difference between the given group and HTF-control group.

**Figure 2:**
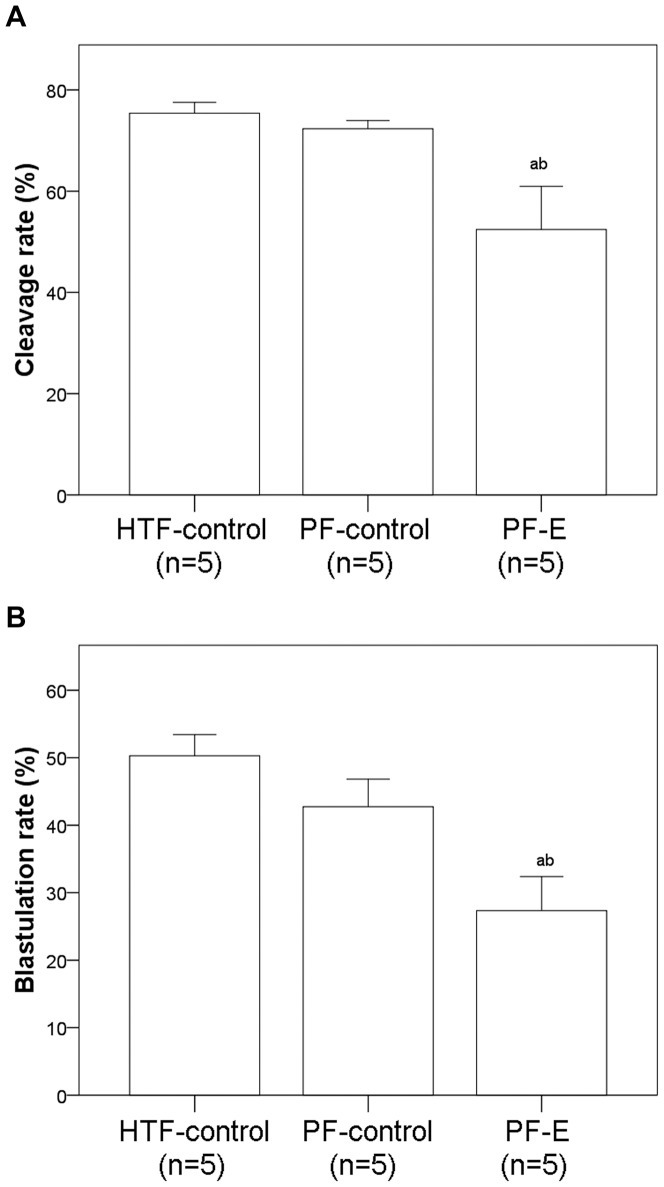
Cleavage (A) and blastulation (B) rates of HTF-control group and groups cultured with peritoneal fluid from patients with endometriosis (PF-E) and those without endometriosis (PF-control). ^a^ Indicates a significant difference between the given group and HTF-control group. ^b^Indicates a significant difference between the PF-control and PF-E groups.

SNP had no effect on the cleavage and blastulation rate at a concentration of 10^−8^ M. However, at concentrations of 10^−6^, 10^−4^, and 10^−3^ M SNP significantly reduced both the cleavage and blastulation rate (Figure 1). Cells cultured with PF from patients with endometriosis had a significantly lower cleavage rate and blastulation rate than both the HTF-control group and cells cultured with PF from patients without endometriosis (*P*<0.05). There was no significant difference in the cleavage rate and blastulation rate between the HTF-control group and the group cultured with fluid from patients without endometriosis (Figure 2).

#### ATP level, mtDNA copies, and ΔΨm

**Figure 3:**
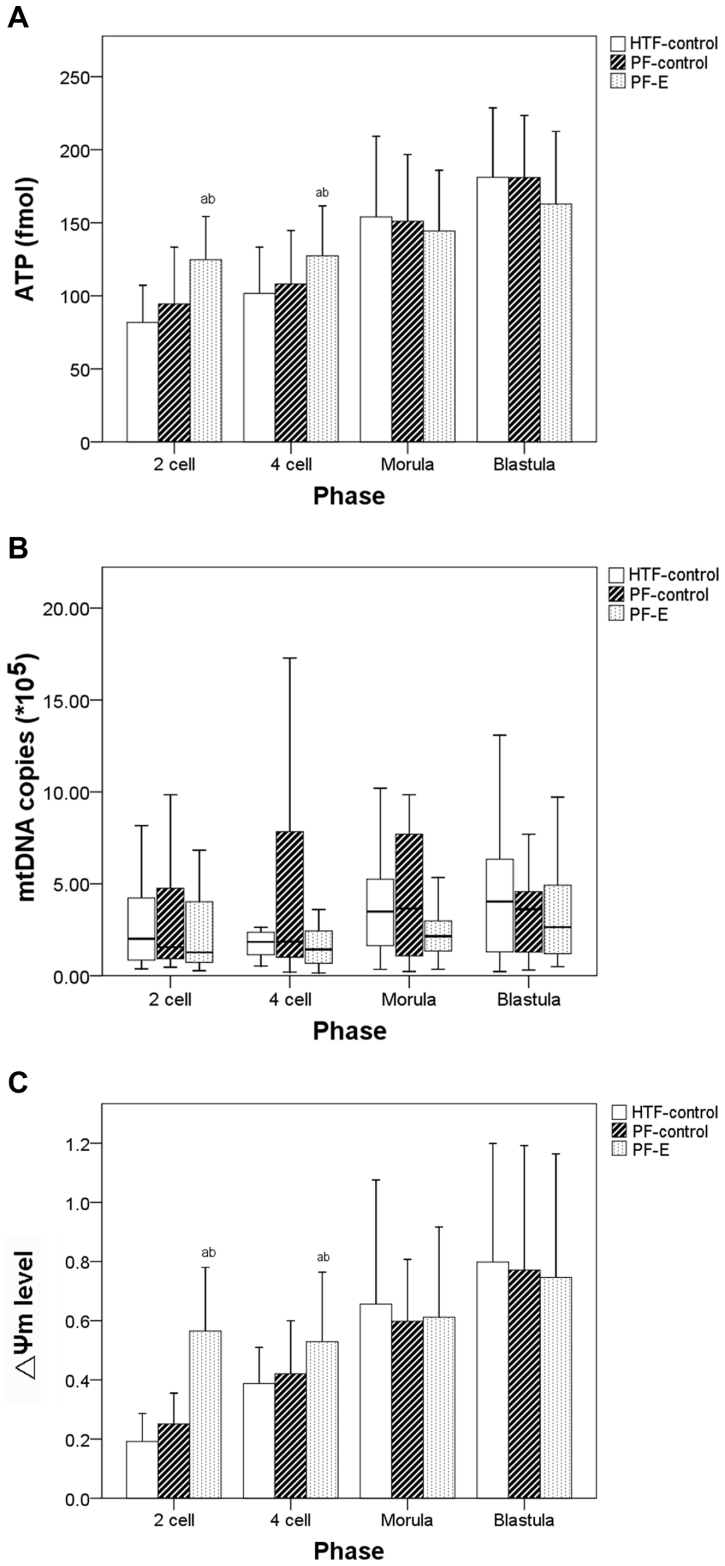
ATP level (A), mtDNA copies (B), and ΔΨ_m_(C) of HTF-control group and groups cultured with peritoneal fluid from patients with endometriosis (PF-E) and those without endometriosis (PF-control) at different phases. ^a^ Indicates a significant difference between the given group and HTF-control group. ^b^Indicates a significant difference between the PF-control and PF-E groups.

**Figure 4:**
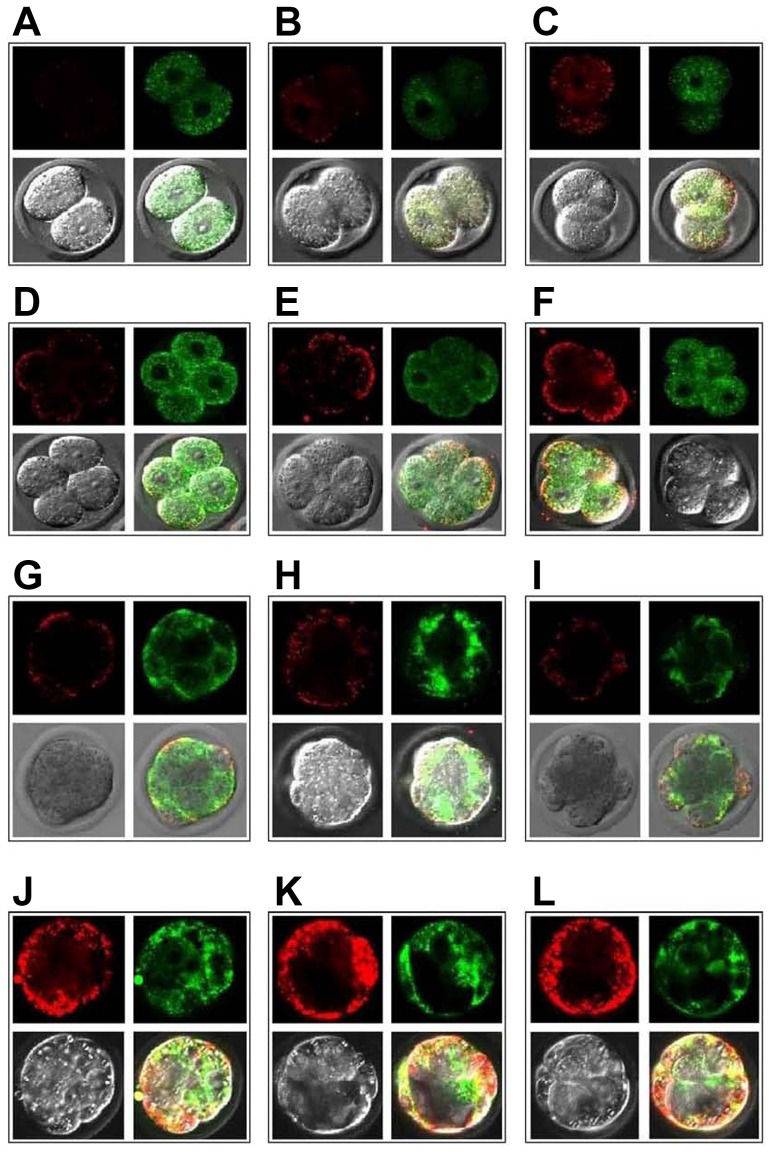
JC-1 staining of different stage early embryos in the HTF-control, PF-control, and PF-E groups. Rows, from top to bottom, correspond to 2-cell, 4-cell, morula, and blastocyst stages, respectively. Columns, from left to right, correspond to the HTF-control (A, D, G, and J), PF-control (B, E, H, and K), and PF-E (C, F, I, and L) groups, respectively.

Embryonic ATP content gradually increased with the embryo development (*P*<0.001, Figure 3A). The PF-E group had a significantly higher ATP level at the 2-cell and 4-cell stages than the PF-control and HTF-control groups (*P*<0.001). In contrast, there was no significant difference among the 3 groups at the morula and blastula stages (Figure 3A). No significant difference in mtDNA copies at the 2-cell, 4-cell, morula, and blastula stages were noted among the 3 groups (Figure 3B). The PF-E group had a significantly higher (Ψm level at the 2-cell and 4-cell stages than the PF-control and HTF-control groups (*P*<0.001). In contrast, there was no significant difference among the 3 groups at morula and blastula stages (Figure 3C). JC-1 staining of different-stage early embryos in the HTF-control, PF-E, and PF-control groups are shown in Figure 4.

#### Electron microscopy of mitochondria

**Figure 5:**
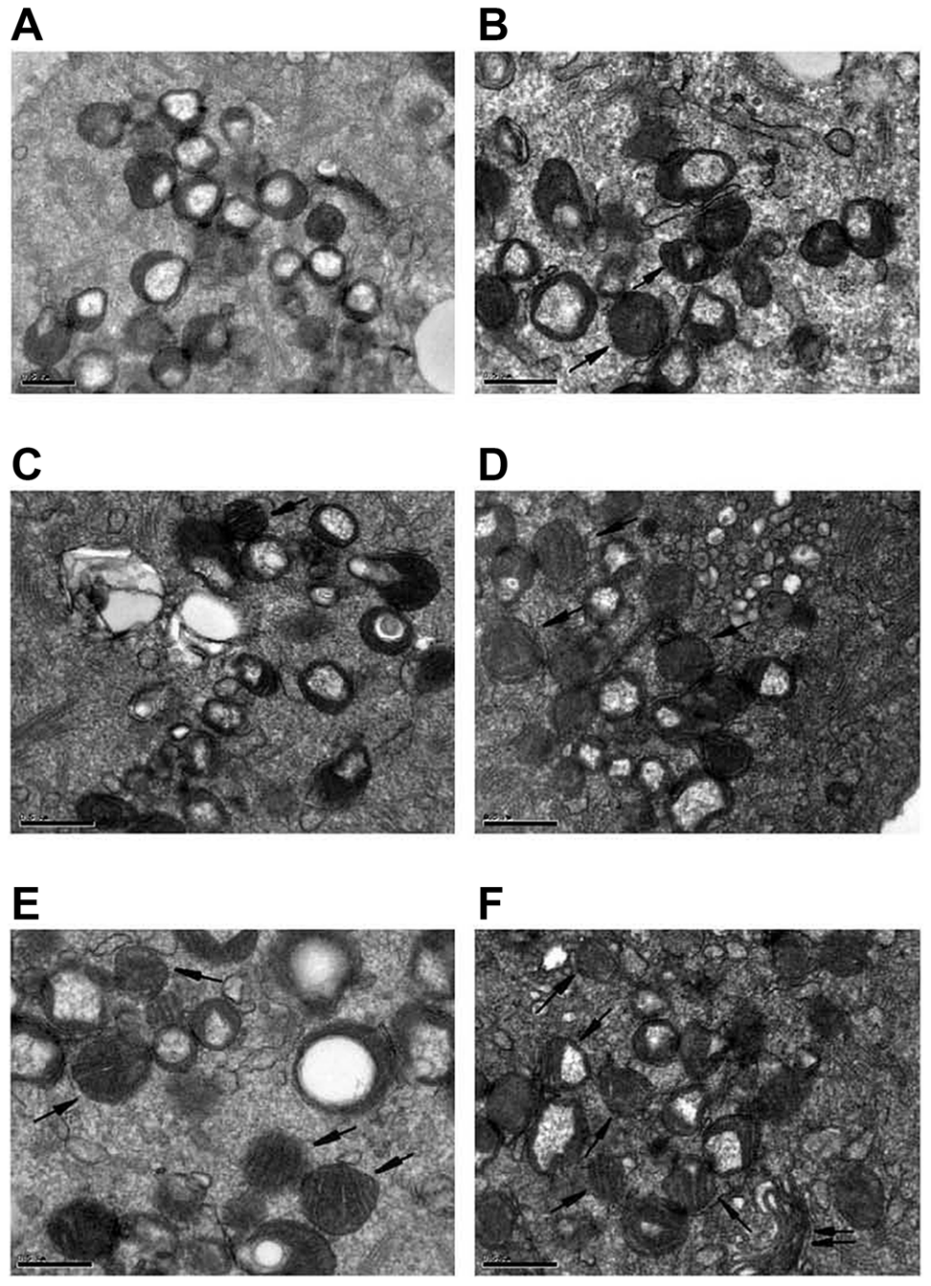
Electron microscopy of 2-cell and 4-cell embryos in the HTF-control, PF-control, and PF-E groups. A) 2-cell embryos in the HTF-control group. The mitochondria were round with few cristae (17,500×). B) 4-cell embryos in the HTF-control group. Several mitochondria with rich cristae were seen (24,000×). C) 2-cell embryos in the PF-control group. Most mitochondria were round with few cristae, and a few mitochondria had a transverse crest (24,000×). D) 4-cell embryos in the PF-control group. Mitochondria with transverse cristae were increased (24,000×). E) 2-cell embryos in the PF-E group. Mitochondria with lamellar transverse cristae were increased significantly (24,000×). F) 4-cell embryos in the PF-E group. Multiple mitochondria with rich transverse cristae were seen (24,000×). (The single arrow indicates mitochondria rich in transverse cristae, and the double arrow indicates the Golgi apparatus.)

Comparisons of the ultramicroscopic structures of embryonic mitochondria are shown in Figure 5. Five 2-cell embryos and five 4-cell embryos were selected from the HTF-control group, the PF-control, and PF-E groups, respectively, for observation. In the HTF-control group embryos the mitochondria were rich and were round with high matrix density and few cristae. Mitochondria in the non-active state and endoplasmic reticulum vesicles formed complexes. In the PF-control group, mitochondria with cristae and multiple vesicular mitochondria were present. In the PF-E group, the number of mitochondria with cristae increased significantly, and the Golgi apparatus appeared.

## Discussion

The results of this study showed that the NO level in the PF of patients with endometriosis was significantly greater than the level in patients with infertility due to tubal factors and in patients with normal fertility. Furthermore, PF from patients with endometriosis inhibited early embryo development and induced functional changes in embryo mitochondria.

A number of studies have suggested that embryo development may be affected by endometriosis. Morcos et al. [10] found that PF of patients with endometriosis inhibited embryonic development in mice, and Gomez-Torres et al. [11] reported that the embryotoxicity of PF from patients with endometriosis is related to the cytokines and lymphocytes in the PF. Mansour et al. [12] found that PF of patients with endometriosis can cause egg microtubule changes, spindle damage, and abnormal meiosis at metaphase II after only 1(hour of incubation. Although Esfandiari did not find any difference in the effects of endometriotic and non-endometriotic PF on early embryo development [13]. Ding et al. [22] recently reported that when oocytes and embryos were cultured in media with PF-E, the fertilization capability of oocytes and the development potential of embryos were decreased. In our previous studies, we showed that oocyte fertilization rate of patients with endometriosis undergoing IVF-ET was lower than that of women with tubal factor infertility. However, no significant differences in implantation rate, pregnancy rate, and abortion rate of IVF-ET between the two groups [17], [18]. That suggested low fertilization rate may caused by toxic effect of endometrial PF to soaked ovary and oocytes , after leaving the PF microenvironment the subsequent formed embryoes can developed the same as in patients with tubal factor infertility. Khamsi et al. [14] reported that although oocytes had contact with endometriotic cyst fluid during oocyte retrieval, the IVF outcome was unaffected. That may be related to the short time of contact between oocytes and endometriotic cyst fluid, and the fact that the oocyte function can recover after it is removed from the previous internal environment.

In this study we found that NO in the PF of patients with endometriosis was significantly higher than in infertile patients without endometriosis due to tubal factor and in control patients with normal fertility. The animal experiments then showed that low levels of NO did not affect embryo development, but high levels did. NO is an active gas radical, which is involved in a variety of biological responses including blood pressure regulation, immunity mediation, and nerve conduction [23]. NO also regulates female reproductive processes including ovulation, implantation, pregnancy maintenance, and delivery regulation [24], [25]. Dong et al. [26] reported that PF NO levels were similar between women with idiopathic infertility and those with endometriosis, and the levels were significantly higher than in control patients with normal fertility, suggesting that high NO maybe the pathogenic factor for idiopathic infertility.

When the NO level is high, NO interacts with other compounds and free radicals, and nitro-based compounds with high oxidation activity are produced which cause damage to proteins, lipids, and DNA [17], [19], [23]. Mitochondria are presumably major target organelles.

To exclude potential toxic effects of PF from endometriosis patients on sperm [27], we used mice 2PN embryos in order to observe the effects on embryonic mitochondria alone. The cleavage and blastocyst formation rates were significantly lower in the endometriosis group, and the ATP content of 2- and 4-cell embryos in the endometriosis group was significantly higher than in the non-endometriosis group, whereas mitochondrial copy number in the 3 groups were not significantly different. Furthermore, the ΔΨm group of 2- and 4-cell embryos was significantly higher in the endometriosis group than in the other 2 groups. These findings suggest that the effect of PF from patients with endometriosis was partially because of the activation of mitochondrial function.

Mitochondria are very sensitive to damage by ROS and RNS [28]. The mitochondria in mammalian embryos do not replicate before embryo implantation; however, mitochondria will experience functional changes to meet the energy needs at different stages, which are characterized by changes in ΔΨm and morphological changes of the organelle. Under normal circumstances, the mitochondria in mature oocytes have a low ΔΨm, and are round with high matrix density and few cristae; they are essentially not active. After a number of cleavages, the mitochondria in a single embryonic cell become fewer and fewer, while cell metabolism gradually increases. After blastomere compaction the embryo enters into a high oxygen consumption state [29], and mitochondria need to produce more ATP in order to maintain embryo growth and metabolism. Normally, the embryonic ΔΨm gradually increases from the 2-cell stage to the morula stage. Round mitochondria with few cristae are gradually replaced by oval mitochondria which are slightly rich in lamellar cristae. From the start of the morula stage, the activity of the mitochondria increases significantly, and the mitochondria become narrower and have more abundant cristae. In the blastocyst stage, metabolism is more vigorous and the ATP content is higher. If the embryo cannot complete the above changes, their development may stagnate [30].

This study demonstrated that in a normal culture environment embryonic ATP content and ΔΨm showed a gradual upward trend from the 2-cell to blastocyst stage. However, the embryonic ATP content and ΔΨm in the endometriosis group showed an increasing trend at the 2-cell and 4-cell stages, ahead of that of the normal culture group. Electron microscopy showed that the number of crest-rich mitochondria in the endometriosis group was increased significantly at the 2-cell and 4-cell stages. The possible reason for these findings is that the PF of patients with endometriosis changed the microenvironment for embryo development, promoting the activation of the embryo mitochondria in advance.

In general, there are 3 stages in which mitochondria regulate the cell responses to stress or a poor environment. 1) In the compensation stage, ROS or RNS improve mitochondrial homeostasis and function (including quantity, quality, and spatial structural changes) through regulating the mitochondrial components or nuclear-related transcription factor. These changes inhibit a further increase of ROS through negative feedback. 2) When the compensation threshold of cells is exceeded, ROS activate cellular inflammatory signaling pathways, leading to cell damage. Mitochondrial damage results in a further increase in ROS, leading to a vicious cycle. In addition, damaged mitochondria can directly stimulate the release of inflammatory substances by inflammasomes. 3) In the final stage, ROS induce mitochondrial permeability transition (MPT), thus triggering the mitochondrial apoptotic program.

In this study, mitochondrial function enhancement in the mouse cleavage-stage embryos can be considered a compensatory enhancement of the embryos in response to an adverse microenvironment. The mitochondrial replication is not triggered in the early embryo, and the number of mitochondria cannot be increased in response to stress, therefore, functional regulation is more important. However, premature mitochondrial function enhancement and the high metabolic state of the cells may in turn increase the production of free radicals, which is not conducive to further growth and development of the embryo. Tarazona et al. [31] observed that in embryos with abnormally high ATP at the 2- to 4-cell stage, the ATP level subsequently declined and cell death occurred in the morula stage [31]. Interestingly, Cho et al. [32] reported that certain mtDNA variants were associated with increased susceptibility to endometriosis.

In summary, the results of this study demonstrated that the NO content in the PF of patients with endometriosis was increased and the PF inhibited early embryo development. The inhibition of embryo development was likely the result of changes to mitochondrial function in early stage of embryo development.

## Supporting Information

Checklist S1 **The ARRIVE Guidelines Checklist (Animal Research: Reporting In Vivo Experiments) had been provided.**
(DOC)
